# Lenalidomide combined with low-dose cyclophosphamide and prednisone modulates Ikaros and Aiolos in lymphocytes, resulting in immunostimulatory effects in lenalidomide-refractory multiple myeloma patients

**DOI:** 10.18632/oncotarget.26131

**Published:** 2018-09-21

**Authors:** Laurens E. Franssen, Inger S. Nijhof, Chad C. Bjorklund, Hsiling Chiu, Ruud Doorn, Jeroen van Velzen, Maarten Emmelot, Berris van Kessel, Mark-David Levin, Gerard M.J. Bos, Annemiek Broijl, Saskia K. Klein, Harry R. Koene, Andries C. Bloem, Aart Beeker, Laura M. Faber, Ellen van der Spek, Reinier Raymakers, Pieter Sonneveld, Sonja Zweegman, Henk M. Lokhorst, Anjan Thakurta, Xiaozhong Qian, Tuna Mutis, Niels W.C.J. van de Donk

**Affiliations:** ^1^ Department of Hematology, VU University Medical Center, Amsterdam, The Netherlands; ^2^ Department of Translational Development, Celgene Corporation, Summit, NJ, USA; ^3^ Laboratory for Translational Immunology, University Medical Center Utrecht, Utrecht, The Netherlands; ^4^ Department of Internal Medicine, Albert Schweitzer Hospital, Dordrecht, The Netherlands; ^5^ Department of Hematology, Maastricht University Medical Center, Maastricht, The Netherlands; ^6^ Department of Hematology, Erasmus Medical Center, Rotterdam, The Netherlands; ^7^ Department of Internal Medicine, Meander Medical Center, Amersfoort, The Netherlands; ^8^ Department of Hematology, St. Antonius Hospital, Nieuwegein, The Netherlands; ^9^ Department of Internal Medicine, Spaarne Hospital, Hoofddorp, The Netherlands; ^10^ Department of Internal Medicine, Rode Kruis Hospital, Beverwijk, The Netherlands; ^11^ Department of Internal Medicine, Rijnstate Hospital, Arnhem, The Netherlands; ^12^ Department of Hematology, University Medical Center Utrecht Cancer Center, Utrecht, The Netherlands

**Keywords:** multiple myeloma, immunomodulation, lenalidomide, refractory, cyclophosphamide

## Abstract

We recently showed that the outcome of multiple myeloma (MM) patients treated in the REPEAT study (evaluation of lenalidomide combined with low-dose cyclophosphamide and prednisone (REP) in lenalidomide-refractory MM) was markedly better than what has been described with cyclophosphamide-prednisone alone. The outcome with REP was not associated with plasma cell Cereblon expression levels, suggesting that the effect of REP treatment may involve mechanisms independent of plasma cell Cereblon-mediated direct anti-tumor activity. We therefore hypothesized that immunomodulatory effects contribute to the anti-MM activity of REP treatment, rather than plasma cell Cereblon-mediated effects. Consequently, we now characterized the effect of REP treatment on immune cell subsets in peripheral blood samples collected on day 1 and 14 of cycle 1, as well as on day 1 of cycle 2. We observed a significant mid-cycle decrease in the Cereblon substrate proteins Ikaros and Aiolos in diverse lymphocyte subsets, which was paralleled by an increase in T-cell activation. These effects were restored to baseline at day one of the second cycle, one week after lenalidomide interruption. *In vitro*, lenalidomide enhanced peripheral blood mononuclear cell-mediated killing of both lenalidomide-sensitive and lenalidomide-resistant MM cells in a co-culture system. These results indicate that the Cereblon-mediated immunomodulatory properties of lenalidomide are maintained in lenalidomide-refractory MM patients and may contribute to immune-mediated killing of MM cells. Therefore, combining lenalidomide with other drugs can have potent effects through immunomodulation, even in patients considered to be lenalidomide-refractory.

## INTRODUCTION

Multiple myeloma (MM) is a malignant disease, characterized by clonal proliferation of plasma cells in the bone marrow. Clinical characteristics include osteolytic bone lesions, hypercalcemia, renal failure, and progressive bone-marrow dysfunction with anemia and other cytopenias. Significant advances have been made during the past decades in the treatment of MM, especially due to the development of immunomodulatory drugs (IMiDs) and proteasome inhibitors [1, 2]. However, patients who become refractory to all available anti-MM agents have a very poor prognosis [3, 4]. The mechanism of action of IMiDs has been shown to be dependent on Cereblon-mediated ubiquitination and subsequent proteasomal degradation of the substrate proteins Ikaros family zinc finger 1 (IKZF1) (Ikaros) and IKZF3 (Aiolos). This leads to downregulation of cMyc and Interferon regulatory factor 4 (IRF4) resulting in growth inhibition and apoptosis of MM cells [5–11]. In addition to these direct anti-tumor effects, Ikaros and Aiolos have been shown to act as repressors of IL-2 transcription. IMiDs induce Cereblon-dependent degradation of Ikaros and Aiolos in immune cells, which increases IL-2 expression, and also enhances the production of other cytokines (IFNγ, IL-4, IL-6, IL-10, IL-13 and GM-CSF), causing activation of both T- and NK-cells [9, 12–17]. Resistance mechanisms to IMiDs however, are still poorly understood. A low expression of Cereblon has been correlated with IMiD resistance [11, 18–21]. as are the presence or development of Cereblon pathway mutations, Cereblon splice variants, or increased activity of the MEK/ERK pathway [22–24]. We have recently reported *in vivo* evidence that plasma cell Cereblon downregulation is one of the characteristics of acquired lenalidomide resistance in patients who were subsequently treated in the REPEAT study [25]. In this study we showed remarkable activity of lenalidomide (Revlimid) combined with continuous low-dose oral cyclophosphamide (Endoxan) and prednisone (REP) in heavily pretreated, lenalidomide-refractory MM patients [26]. The outcome of REP treatment was better than what has been described with cyclophosphamide-prednisone alone, suggesting synergistic effects of the lenalidomide-cyclophosphamide combination [27, 28]. Although several studies have shown a correlation between Cereblon expression in MM cells and clinical outcomes of IMiD based therapy [18–20, 29], the outcome with REP treatment was not associated with plasma cell Cereblon expression levels, suggesting that the effect of REP treatment may involve mechanisms independent of plasma cell Cereblon-mediated direct anti-tumor activity [25]. We therefore hypothesized that immunomodulatory effects contribute to the anti-MM activity of REP treatment, rather than plasma cell Cereblon-mediated effects. Consequently, we here analyzed the frequency and activity of lymphocyte subsets from patients treated in the REPEAT study, to characterize the effect of REP treatment on the immune system of lenalidomide-refractory MM patients.

## RESULTS

### Ikaros and Aiolos can still be modulated in lymphocytes from lenalidomide-refractory patients during REP treatment

**Figure 1:**
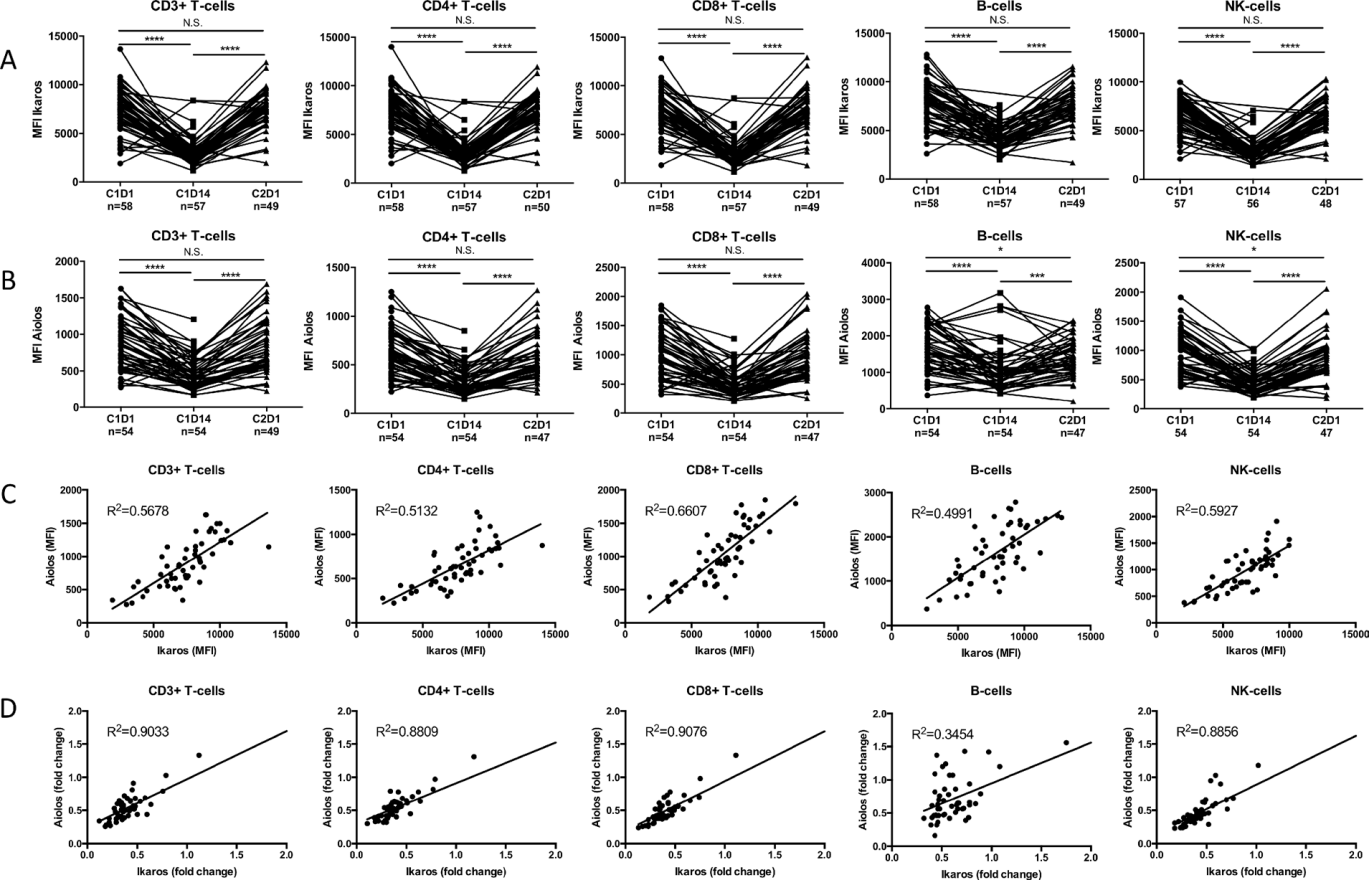
During REP treatment, Ikaros and Aiolos levels can be modulated in lymphocytes from lenalidomide-refractory patients PBMCs obtained at the start of cycle 1 (C1D1), mid-cycle (C1D14) and start of cycle 2 (C2D1) were stained for CD3, CD4, CD8, CD19 and CD56 to identify the different lymphocyte subsets. Lymphocytes were then stained for intracellular Ikaros (**A**) and Aiolos (**B**) expression and analyzed by flow cytometry. (**C**) Correlation between baseline Ikaros and Aiolos expression levels in the different lymphocyte subsets. (**D**) Correlation between fold change from C1D1 to C1D14 in Ikaros and Aiolos expression in the different lymphocyte subsets. *P-*values were calculated using the Wilcoxon matched pairs, signed rank test. ^***^P < 0.001, ^****^P < 0.0001, *N.S.* not significant.

Sixty-four lenalidomide-refractory MM patients were treated with REP in the phase 2 part of the REPEAT study. Lenalidomide was administered on days 1 to 21 of a 28-day cycle, and cyclophosphamide and prednisone were given continuously. PBMCs obtained at the start of REP treatment, at day 14 and at day 1 of cycle 2 (before the administration of anti-MM agents) were analyzed for Ikaros and Aiolos expression using flow cytometry. The results revealed that two weeks of REP treatment caused a significant decrease in expression of Ikaros and Aiolos both in CD4^+^ T-cells (median decrease: 63% and 46%, resp.) and CD8^+^ T-cells (median decrease: 63% and 55%, resp.), NK-cells (median decrease: 59% and 57%, resp.), and B-cells (median decrease: 46% and 37%, resp.) (Figure 1A, 1B). There was a significant correlation between baseline Ikaros and Aiolos expression (Figure 1C; Pearson correlation: CD3^+^ T-cells: R^2^ 0.57, *P <* 0.001; CD4^+^ T-cells: R^2^ 0.51, *P* < 0.001; CD8^+^ T-cells: R^2^ 0.67, *P* < 0.001; NK-cells: R^2^ 0.59, *P <* 0.001; B-cells: R^2^ 0.50, *P <* 0.001). Similarly, a significant correlation was observed between the extent of Ikaros and Aiolos downregulation (Figure 1D; Pearson correlation: CD3^+^ T-cells: R^2^ 0.90, *P <* 0.001; CD4^+^ T-cells: R^2^ 0.88, *P* < 0.001; CD8^+^ T-cells: R^2^ 0.91, *P* < 0.001; NK-cells: R^2^ 0.89, *P <* 0.001; B-cells: R^2^ 0.342, *P <* 0.001) in all these immune cell subsets. Ikaros and Aiolos expression levels were restored to baseline levels at day 28, which was after one week without lenalidomide treatment. These results indicated that in these lenalidomide-refractory patients, Ikaros and Aiolos expression in lymphocytes can still be modulated by REP treatment. Nonetheless, the baseline expression of Ikaros and Aiolos levels or the extent of Aiolos/Ikaros reduction in these immune cells did not show a significant correlation with response, PFS or OS following REP treatment.

### REP treatment induces T-cell activation

**Figure 2:**
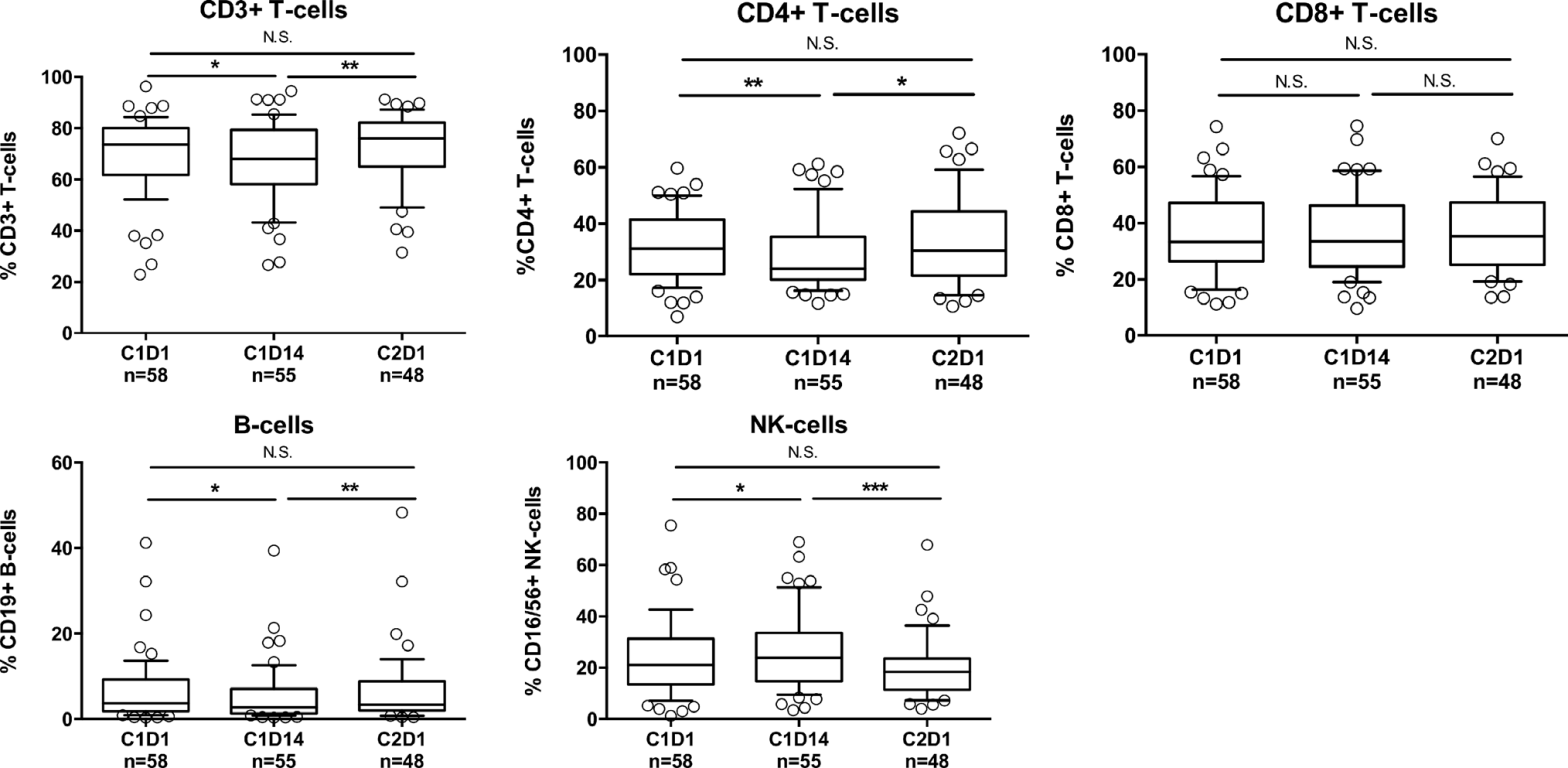
Changes in lymphocyte frequencies following REP treatment Frequencies of T-cells, CD4^+^ T-cells, CD8^+^ T-cells, B-cells, and NK-cells before start of REP treatment (C1D1), mid-cycle (C1D14) and at the start of cycle 2 (C2D1). Boxes represent first and third quartile with median value, and error bars represent p10 to p90. *P-*values were calculated using the Wilcoxon matched pairs, signed rank test, or paired *T*-test, depending on the distribution. ^*^P < 0.05, ^**^P < 0.01, ^***^P < 0.001, *N.S.* not significant.

**Figure 3:**
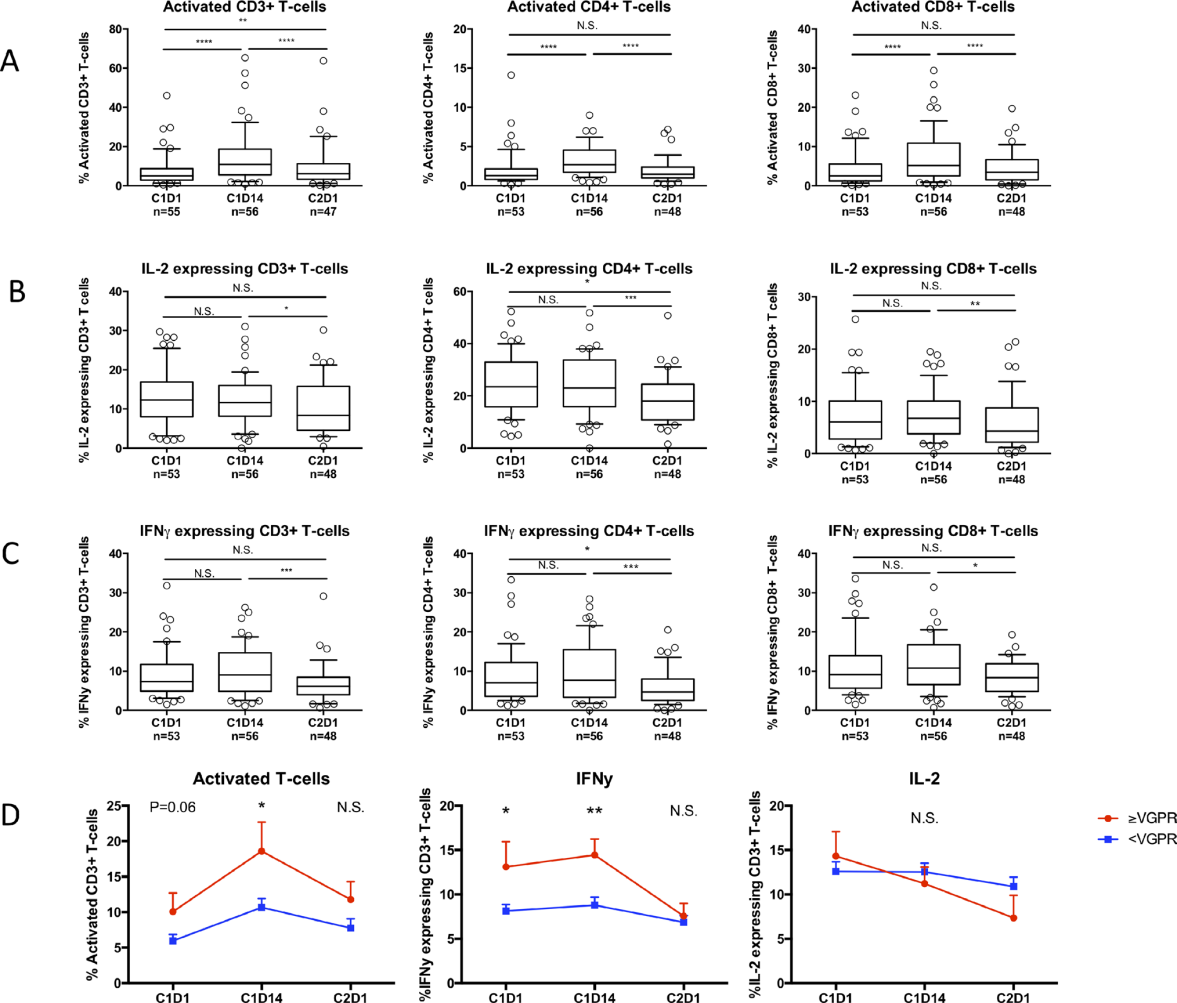
Effects of REP treatment on T-cell activation and cytokine production in lenalidomide-refractory patients PBMCs were obtained before start of cycle 1 (C1D1), mid-cycle (C1D14) and before start of cycle 2 (C2D1). (**A**) Change in frequencies of activated (HLA-DR^+^) T-cells during REP treatment. (**B**, **C**) Changes in expression of IL-2 (**B**) and IFNg (**C**) in T-cells during REP treatment. Boxes represent first and third quartile with median value, and error bars represent p10 to p90. (**D**) Change in activated (left panel), IFNg producing (middle panel) and IL-2 producing (right panel) T-cells in patients with a response ≥VGPR (red) versus <VGPR (blue) during REP treatment. Shown are mean ± SEM. *P*-values were calculated using the Wilcoxon matched pairs, signed rank tests (**A**, **B**, **C**) and *T*-tests (**D**). ^*^P < 0.05, ^**^P < 0.01, ^***^P < 0.001, ^****^P < 0.0001, *N.S.* not significant.

Next, we investigated the impact of REP treatment in lenalidomide-refractory patients on the frequency of peripheral blood lymphocyte subsets and on T-cell activation status (HLA-DR expression) and cytokine production (IFNγ and IL-2). Frequencies of total CD3^+^ T-cells, CD4^+^ T-cells, and CD19^+^ B-cells slightly decreased during the first 14 days, while NK-cells increased (Figure 2). These levels returned to baseline at day 1 of cycle 2. Levels of CD8^+^ T-cells did not change. However, the observed changes were relatively small. There was a significant increase in the frequency of activated CD4^+^ and CD8^+^ T-cells mid-cycle, which decreased to baseline at the start of cycle 2 (Figure 3A). The production of IFNγ and IL-2 by CD4^+^ and CD8^+^ T-cells did not change significantly from baseline to mid-cycle. However, there was a significant reduction in IFNγ and IL-2 production at the start of cycle 2 compared to mid-cycle levels, which is after one week without lenalidomide treatment (Figure 3B, 3C). This indicates that in lenalidomide-refractory patients, Ikaros and Aiolos degradation in immune cells is associated with an increase in activated T-cells. Although there was no correlation between overall response (≥partial response (PR)) and frequency of activated T-cells or IFNγ/IL-2 production by T-cells, we observed that patients who achieved at least a very good partial response (VGPR) (*n* = 13) had a significantly higher percentage of mid-cycle activated CD3^+^ T-cells (18.58% vs. 10.68%, *P* = 0.01) and a significantly higher baseline (IFNγ^+^ CD3^+^ 13.11% vs. 8.14%, *P* = 0.02) and mid-cycle IFNg production by T-cells (IFNγ^+^ CD3^+^ 14.43% vs. 8.80%, *P* = 0.006) when compared to patients with less than VGPR (*n* = 45) (Figure 3D), suggesting the contribution of T-cell immune responses in achieving ≥VGPR.

### Regulatory T-cells and PD-1 expression on NK-cells increase during REP treatment

**Figure 4:**
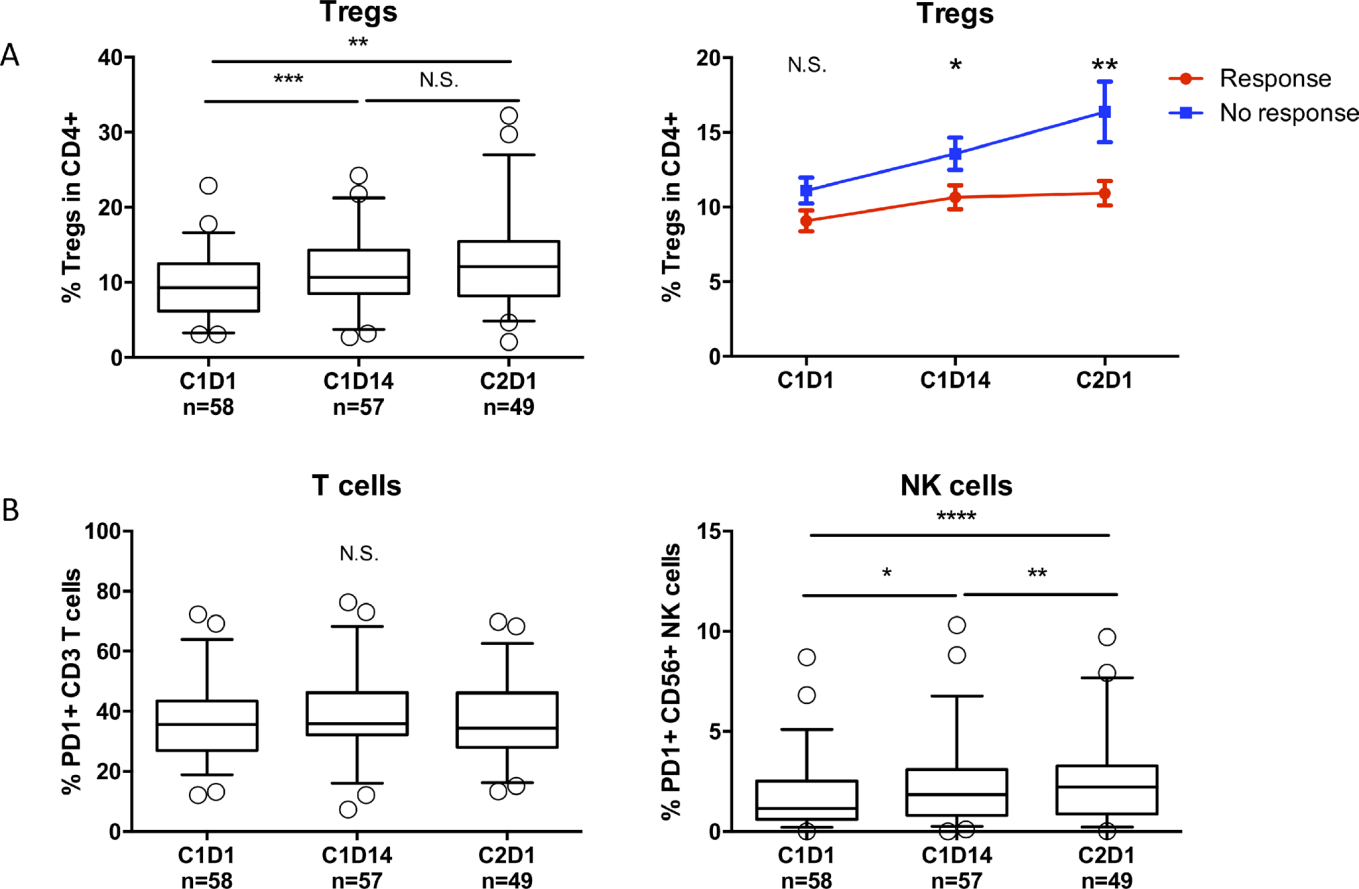
REP treatment causes an increase in regulatory T-cells and increased PD-1 expression on NK-cells (**A**) Left panel: Frequencies of regulatory T-cells during REP treatment. Boxes represent first and third quartile with median value, and error bars represent p10 to p90. Right panel: Change in regulatory T-cell frequencies in responding patients (≥PR, red line) versus non-responding patients (<PR, blue line) during REP treatment. Shown are mean ± SEM. (**B**) Frequencies of PD-1+ T-cells (left panel) and NK-cells (right panel) during REP treatment. Boxes represent first and third quartile with median value, and error bars represent p10 to p90. *P-*values were calculated using the Wilcoxon matched pairs, signed rank tests (**A**, left panel and **B**) and T-tests (**A**, right panel). ^*^P < 0.05, ^**^P < 0.01, ^***^P < 0.001, ^****^P < 0.0001, *N.S.* not significant.

Several reports have shown that lenalidomide induces an increase in regulatory T-cells (Tregs), which may hamper the beneficial effect of an increased frequency of activated T-cells [30–37]. On the other hand, metronomic dosing of cyclophosphamide has been shown to deplete regulatory T-cells [38–43]. Therefore, we hypothesized that the addition of cyclophosphamide to lenalidomide treatment may prevent the lenalidomide-induced increase in Tregs. However, similar to earlier studies performed with lenalidomide alone, we observed a significant increase in Tregs during the first REP cycle (Figure 4A, left panel). Interestingly though, the increase in Tregs was more pronounced in non-responding patients (<PR) as compared to responding patients. Furthermore, non-responders showed an ongoing increase in Tregs, while responders showed equal frequencies of Tregs from mid-cycle to the start of cycle 2 (Figure 4A, right panel). These differences were not observed when response was defined as ≥VGPR. PD-1 expression on CD3^+^ T-cells and NK-cells was also analyzed. We observed no changes in PD-1 expression on T-cells, and a modest increase in PD-1 expression on NK-cells (Figure 4B).

### Lenalidomide enhances PBMC-mediated killing of both lenalidomide-sensitive and lenalidomide-resistant MM cells

**Figure 5:**
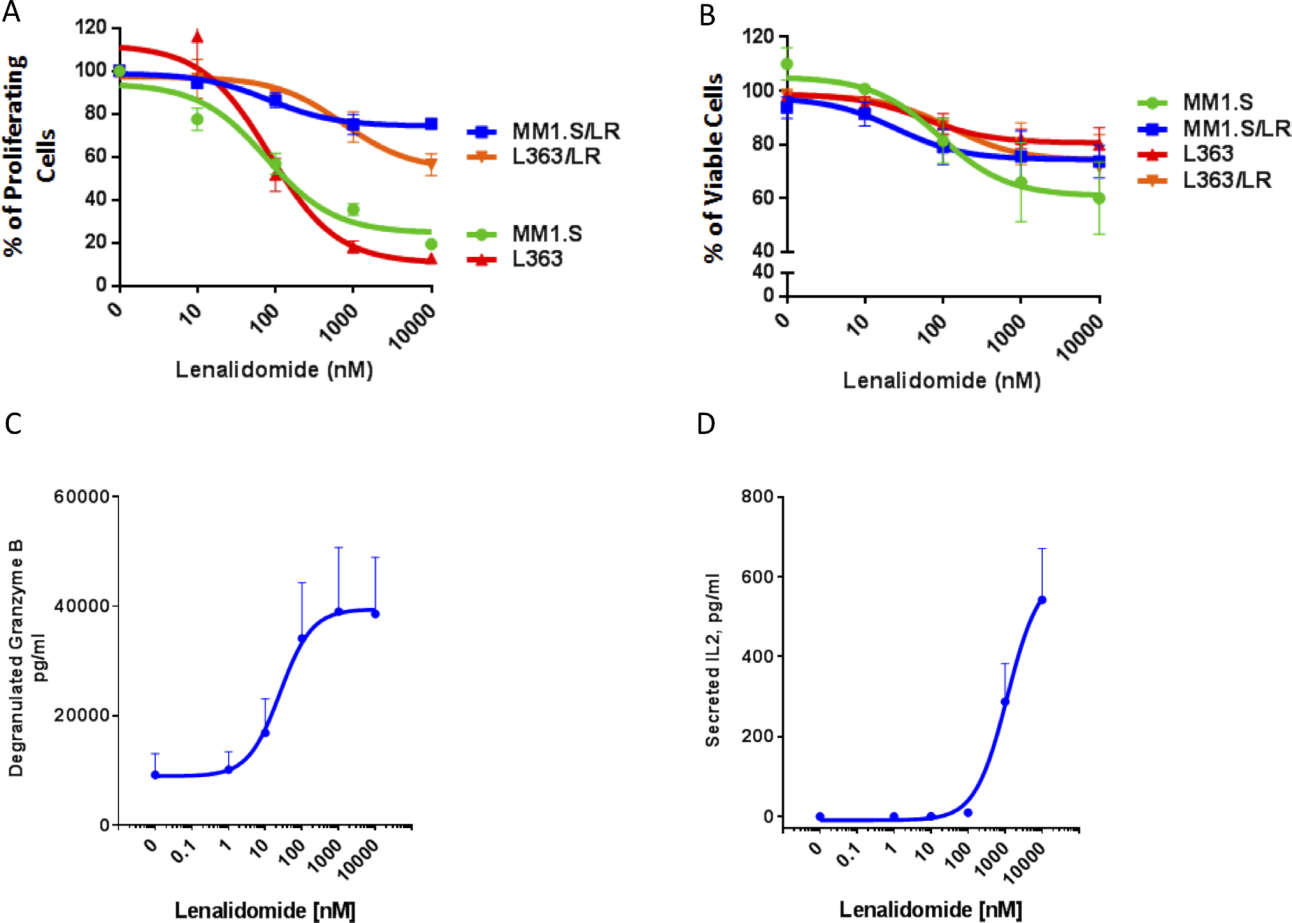
Lenalidomide enhances PBMC-mediated killing of both lenalidomide-sensitive and lenalidomide-resistant MM cells (**A**) ^3^H-thymidine incorporation in lenalidomide-sensitive MM1.S and L363 and their lenalidomide-resistant progeny cells (MM1.S/LR, L363/LR) following treatment with either vehicle control or lenalidomide (10–10000 nM). The results are presented as percent of the vehicle control. (**B**) The portion of viable MM cells as percent of the vehicle control in a co-culture assay with lenalidomide-pretreated PBMCs obtained from a healthy donor. CFSE-labeled lenalidomide-sensitive MM1.S and L363 and their lenalidomide-resistant progeny (MM1.S/LR, L363/LR) cells were cultured for 4 hours at 3:1 ratio with PBMCs, which were pretreated for 72 hours with muromonab-CD3 and lenalidomide (10–10000 nM). The viable MM cells were identified by Annexin-V/To-Pro-3 negative staining by flow cytometry. (**C**) ELISA measurement of granzyme B levels in the supernatant of the PBMC/MM cell co-culture as depicted in panel (**B**). (**D**) Secreted IL-2 from PBMCs that were treated with lenalidomide (0–10000 nM) for 72 hours. IL-2 levels were measured by ELISA from the supernatant of PBMCs that were subsequently used for the co-culture experiment as depicted in panel (**B**). Negative values were set at 0 (for 4 data points, their value in the ELISA readout was lower than the reference value 0). All figures shown are representatives of *n* = 3 experiments.

To further investigate the immune-mediated effects of lenalidomide in lenalidomide-refractory MM, we used two lenalidomide-sensitive MM cell lines (MM1.S and L363) and generated lenalidomide-resistant progeny cell lines (MM1S/LR and L363/LR) as previously described (Figure 5A) [27]. To eliminate direct effects of lenalidomide on the MM cells, PBMCs from healthy donors were pretreated for 72 hours with either vehicle control or lenalidomide in different concentrations, washed, and then co-cultured with the lenalidomide-sensitive and the lenalidomide-resistant MM cell lines for 4 hours. Lenalidomide was not cytotoxic to PBMCs (Supplementary Figure 2). Flow-cytometric analysis of MM cell viability after 4 hours revealed that lenalidomide enhanced the PBMC-mediated killing of both lenalidomide-sensitive and lenalidomide-resistant cell lines (Figure 5B), with increased granzyme B release in the co-culture system (Figure 5C). Furthermore, lenalidomide treatment induced profound IL-2 secretion by PBMCs (Figure 5D). Altogether, these results indicate that lenalidomide induces an activation of the immune-system, capable of killing MM cells, independent of their lenalidomide-sensitivity.

## DISCUSSION

In the present study, we show in peripheral blood samples collected from 64 lenalidomide-refractory MM patients that lenalidomide combined with low-dose cyclophosphamide and prednisone (REP) was capable of inducing degradation of Ikaros and Aiolos in T, NK and B-cells. This degradation was associated with an increase in NK-cells and activated T-cells. Due to limited availability of patient PBMC samples, we were not able to analyze the effect of REP treatment on NK-cell activation. Furthermore, we show that lenalidomide enhances PBMC-mediated killing of both lenalidomide-sensitive and lenalidomide-refractory MM cells. Although we observed statistically significant changes in the frequencies of different lymphocyte subsets and expression of PD-1 on NK-cells during REP treatment, these changes were relatively small and it is therefore currently unclear to what extent these effects are biologically relevant. Our results are consistent with a recent study, showing similar immune-activating effects of pomalidomide-dexamethasone in lenalidomide-refractory patients [16, 22]. However, our observation that lenalidomide itself retains its immunomodulatory capacity despite the presence of lenalidomide-refractory MM has not been described before. Our observation that the immunomodulatory properties of lenalidomide are maintained despite a clinical lenalidomide-refractory status can have implications for other combination therapies. In fact, pre-clinical and clinical studies have already shown promising results combining lenalidomide and therapeutic antibodies such as isatuximab and daratumumab (anti-CD38) in lenalidomide-refractory MM patients [45–48].

In our study, there was no significant increase in IL-2 and IFNγ production by T-cells at day 14 of cycle 1 compared to baseline levels of these cytokines. However, *in vitro* experiments indicate that lenalidomide-induced degradation of Ikaros and Aiolos already occurs after a 3–6 hour incubation, [14] and an increase in cytokine production can be measured *ex vivo* after 7 days [16]. In addition, our own *in vitro* data show a profound increase in IL-2 production by PBMCs after a 72-hour incubation with lenalidomide (Figure 5C). Therefore, it is possible that in our patient samples obtained at day 14 after start of REP treatment, the maximum effect has passed or the cytokines have already been consumed by the activated immune system. The observation that there was a significant reduction of IL-2 and IFNγ production in T-cells after one week without lenalidomide treatment (while cyclophosphamide and prednisone were given continuously), suggests a lenalidomide-mediated stimulation of cytokine production in these patients.

The decrease in activation status and cytokine production after one week of stopping lenalidomide may suggest that continuous lenalidomide administration, rather than intermittent dosing on days 1–21 of 28-day cycles, could be favorable. However, a previous report described the randomized comparison between continuous pomalidomide and 21/28 days pomalidomide administration, both combined with dexamethasone, in relapsed MM patients [16]. Both treatment arms showed an initial decrease in Ikaros expression in T- and NK-cells, but also in both arms this returned to baseline levels at day 28 (although less pronounced in the continuous treatment arm). This suggests that with continuous treatment, the effects on Ikaros and Aiolos are probably reversed after some time, which may induce an IMiD resistant phenotype of the immune system. Moreover, continuous treatment is probably associated with increased toxicity, when compared to intermittent dosing.

Despite the fact that all patients included in the REPEAT study were lenalidomide refractory and 66% were also bortezomib refractory, REP treatment showed a remarkable overall response rate (ORR) of 67% and a median PFS and OS of 12.1 and 29.0 months respectively [26]. This is markedly higher than what has been described with cyclophosphamide-prednisone treatment alone in relapsed/refractory MM, suggesting synergistic effects of the lenalidomide-cyclophosphamide combination [27, 28]. While we could not exactly determine the nature of such a synergy, our results provide some insights for future studies. It seems unlikely that cyclophosphamide had a direct effect on Ikaros/Aiolos, because the levels of Aiolos and Ikaros returned rapidly to baseline levels within one week without lenalidomide, but with continuous cyclophosphamide. Therefore, the combined effects should have more complex mechanisms. Continuous low-dose cyclophosphamide has been shown to mediate not only direct anti-tumor activity, but also improves anti-tumor immunity via depletion of regulatory T-cells [38–43, 49–51]. When combined with lenalidomide, the depletion of Tregs may not be clearly visible, because it is known that lenalidomide causes an increase in the frequencies of regulatory T-cells [30, 40, 52–55], probably as a compensatory mechanism for the activated T-cell response. Indeed, we observed only a relatively small increase in regulatory T-cell frequencies during REP treatment. Moreover, the increase in responding patients was less pronounced compared to non-responding patients and showed a stabilization between day 14 and day 28 in the first cycle of REP treatment. In addition, patients achieving at least VGPR had a higher percentage of mid-cycle activated T-cells and also higher baseline and mid-cycle IFNγ-producing T-cells when compared to patients with less than VGPR, also suggesting the contribution of an improved immune response in mediating the anti-MM activity of the REP regimen.

Importantly, due to the phase 2 design of the REPEAT study, it is not possible to define the relative importance of each individual drug to the observed immunomodulatory effects. It may also be possible that the combined effects of lenalidomide and cyclophosphamide are due to their known suppressive effects on angiogenesis and inhibition of MM cell adhesion to stromal cells, which have not been analyzed in this study [50, 56].

In conclusion, we show that in lenalidomide-refractory patients, Ikaros and Aiolos expression levels can be modulated in immune cells during REP treatment, which is paralleled by an increase in activated T-cells. In addition, lenalidomide enhances the PBMC-mediated killing of lenalidomide-refractory MM cells *in vitro*. These results indicate that combining lenalidomide with other immunotherapeutic drugs, such as monoclonal antibodies, can still have potent effects through immunomodulation, even in patients considered to be lenalidomide-refractory.

## MATERIALS AND METHODS

### Patients

The study population consisted of the patients in the phase 2 part of the REPEAT study that has been described in detail previously. [26] Briefly, the REPEAT study was a prospective, investigator-initiated, nonrandomized, multicenter, open-label, phase 1 dose-finding trial, followed by a phase 2 expansion at the recommended dose level (RDL) to evaluate the safety, tolerability, and efficacy of lenalidomide, low-dose oral cyclophosphamide and prednisone (REP) in lenalidomide-refractory MM patients. The maximum tolerated dose (MTD) in phase 1 was the RDL for the patients treated in the phase 2 part of the REPEAT study (25 mg lenalidomide (days 1–21/28 days), combined with continuous low-dose oral cyclophosphamide 50 mg/day and prednisone 20 mg/day). Patients were eligible to participate if they had lenalidomide-refractory disease following at least 1 prior therapy. Lenalidomide-refractory MM was defined as progressive disease during therapy, no response (less than PR) to prior lenalidomide-containing therapy, or progression within 60 days of discontinuation from lenalidomide-containing regimens, according to the International Myeloma Working Group criteria. [3] REP therapy was given until disease progression. Response was defined according to the “International Myeloma Working Group Criteria for response and minimal residual disease assessment” [3]. The study was approved by the institutional medical ethical committee in each participating center in accordance with the declaration of Helsinki. All participants provided written informed consent. The trial was registered at https://www.clinicaltrials.gov/ as #NCT01352338. In this analysis, only patients treated at the RDL were included (*n* = 64). Patient characteristics are shown in Table 1.

**Table 1:** Patient characteristics

Characteristic	Total (*n* = 64)
Median age, y (range)	65 (43–82)
Sex, male, *n* (%)	43 (67)
**Response, *n* (%)**	
≥PR	43 (67)
≥VGPR	15 (23)
**Type of monoclonal heavy chain, *n* (%)**	
IgG	37 (57.8)
IgA	8 (12.5)
IgD	0 (0)
Light chain only	19 (29.7)
**Type of light chain, *n* (%)**	
Kappa	42 (65.6)
Lambda	22 (34.4)
Median time from diagnosis until enrollment REPEAT study in months (range)	51.5 (5.37–673)
Prior lines of therapy, median (range)	3 (1–6)
**Prior therapies, *n* (%)**	
Lenalidomide	64 (100)
Bortezomib	53 (82.8)
Thalidomide	38 (59.4)
Cyclophosphamide	25 (39.1)
Autologous stem cell transplantation (HDM)	35 (54.7)
Oral melphalan	27 (42.2)
Allogeneic stem cell transplantation	4 (6.3)
**Previous lenalidomide, *n* (%)**	
Refractory^*^	64 (100)
Progression while on lenalidomide-based therapy^**^	60 (93.8)
No response during prior lenalidomide-based therapy^***^	1 (1.6)
Progressive disease within 60 days after stopping lenalidomide-based therapy^****^	3 (4.7)
Primary lenalidomide refractory#, *n* (%)	14 (21.9)
REP directly after development of lenalidomide-refractory disease, *n* (%)	53 (82.8)
Lenalidomide and bortezomib double refractory^*^, *n* (%)	38 (59.4)
**Cytogenetic abnormalities, *n* (%)**	
High risk##	23 (35.9)
Standard risk	19 (29.7)
Not available	22 (34.4)

Abbreviations: *HDM*, high-dose melphalan; *PR,* partial response; *VGPR,* very good partial response.

^*^Refractory disease is defined as progressive disease during therapy, no response (< PR), or progressive disease within 60 days of stopping treatment, according to the International Uniform Response Criteria for Multiple Myeloma.

^**^Forty-nine patients progressed while receiving lenalidomide (25 mg)-dexamethasone, 2 while receiving lenalidomide, bortezomib and dexamethasone, 1 while receiving MPR (10 mg lenalidomide), and 8 while receiving lenalidomide maintenance therapy (10 mg).

^***^One patient received lenalidomide (25 mg)-dexamethasone.

^****^Two patients received lenalidomide (25 mg)-dexamethasone, and 1 patient received 10mg lenalidomide in MPR.

^#^Primary lenalidomide-refractory was defined as a best response on previous lenalidomide treatment of < PR.

^##^High-risk cytogenetic abnormalities were defined by the presence of t(4;14), t(14;16), del(17p), and/or ampl(1q) as determined by FISH analysis on purified MM cells before start of REP treatment.

### Immune-monitoring

Peripheral blood samples were collected at day 1 and 14 of cycle 1, as well as on day 1 of cycle 2 of REP treatment (before administration of the anti-MM agents). To identify the different immune cell subsets, nucleated cells of whole blood were stained with fluorochrome-conjugated antibodies after lysis of red blood cells (Lysing solution, BD Biosciences). The following antibodies were used: CD3-FITC, CD4-APC, CD8-PE and CD45-PerCP (T-cells), CD3-FITC, CD16/56-PE, CD45-PerCP and CD19-APC (NK-cells and B-cells), HLA-DR-PE, CD3-PerCP and CD8-APC (activated T-cells) (all antibodies from BD biosciences). Activated T-cells were defined as the percentage of T-cells expressing HLA-DR.

Flow cytometry was performed using a FACS Canto II or LSR Fortessa Analyzer (BD Biosciences) and data were analyzed using FACSDIVA v8.0.1 software (BD Biosciences). Additional analyses were performed with cryopreserved peripheral blood mononuclear cells (PBMCs), isolated by Ficoll density centrifugation. The LIVE/DEAD^®^ Fixable Dead Cell Staining kit (Thermofisher Scientific) was used to determine the viability of the cells prior to the fixation and permeabilization and subsequent intracellular staining with antibodies against Ikaros, Aiolos, interferon-γ (IFNγ) and interleukin-2 (IL-2). The FOXP3/Transcription Factor Staining Buffer Set (eBioscience) was used for the fixation and intracellular staining according to the manufacturer's protocol. To measure intracellular Ikaros and Aiolos expression, PBMCs were labeled with appropriate surface markers to identify T, B and NK-cells and stained intracellularly for Ikaros and Aiolos using PE-conjugated antibodies (BD Pharmingen). Cytokine production (IL-2 and IFNγ) of T-cells was measured after stimulating PBMCs with CD3/CD28 Human T-activator Beads (Dynabeads^®^) in a 1:1 ratio for 5 hours at 37°C in the presence of an inhibitor of intracellular protein transport (Brefeldin A, eBioscience). For the gating strategy used in the flow cytometry analysis to detect activated T-cells, T-cells producing IL-2 or IFNγ and regulatory T-cells, see Supplementary Figure 1A–1C.

### Generation of lenalidomide-resistant cell lines

The human MM-derived lines MM1.S and L363 (ATCC, Manassas, VA, USA) were maintained and routinely tested for mycoplasma. Acquired lenalidomide-resistant cell lines were generated as previously described and cultured in the presence of 10 μM lenalidomide [44]. Lenalidomide was removed from culture for a minimum of 5 days prior to any *in vitro* experiments described in the manuscript.

### PBMC co-culture with MM cells

PBMCs were isolated from buffy coats obtained from healthy donors via ficoll separation. Isolated PBMCs were treated with solvent control or lenalidomide for 1 h prior to stimulation with 3 mg/ml plate-bound anti-CD3 (OKT3; eBiosciences). After 72 hrs of culture, supernatants were collected for IL-2 ELISA (R & D Systems). The PBMCs were washed and subsequently co-cultured with carboxyfluorescein succinimidyl ester (CSFE, Invitrogen)-labeled MM cells at 3:1 ratio for 4 hours; supernatants were collected again at the end of the co-culture for Granzyme B release ELISA (Biolegend). The cells were then washed and stained with Annexin-V-PE (BD Biosciences) and To-Pro3-APC (Invitrogen) according to manufacturer's protocol. Live target cells were gated as CFSE**^+^**, Annexin-V**^−^**, and Topro**^−^** singlets by flow cytometry.

### IL-2 and Granzyme B ELISA

IL-2 and Granzyme B level in supernatant were determined using ELISA kit, following manufacturer's protocol from R&D and Biolegend, respectively.

### Statistics

Continuous variables were analyzed using Wilcoxon matched-pairs test or a (paired) T-test depending on the distribution levels. Differences in categorical variables were determined with the Fisher's exact test for two by two tables and otherwise with the Pearson's χ^2^ test. Results are expressed as 2-tailed *P* values. A level of *P* < 0.05 was considered significant. Calculations were performed in SPSS version 20.0.0 (IBM SPSS Inc., Armonk, NY, USA) and GraphPad Prism version 5.03 (GraphPad Software Inc., La Jolla, CA, USA).

## SUPPLEMENTARY MATERIALS FIGURES


