# Supplementary material for: Lenalidomide combined with low-dose cyclophosphamide and prednisone modulates Ikaros and Aiolos in lymphocytes, resulting in immunostimulatory effects in lenalidomide-refractory multiple myeloma patients
Source: Oncotarget. 2018 Sep 21;9(74):34009–21. doi: 10.18632/oncotarget.26131 (PMC6188055; doi:10.18632/oncotarget.26131)
Supplement: Supplementary file 1 [file oncotarget-09-34009-s001.pdf]

Lenalidomide combined with low-dose cyclophosphamide and prednisone modulates Ikaros and Aiolos in lymphocytes, resulting in immunostimulatory effects in lenalidomide-refractory multiple myeloma patients

SUPPLEMENTARY MATERIALS

A Activated T-cells

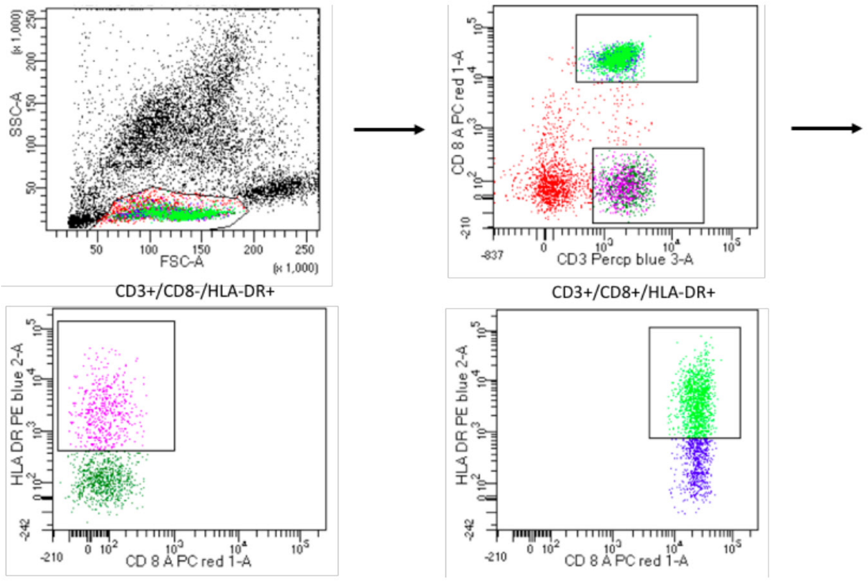

B IFNγ and IL-2 expression in T-cell subsets

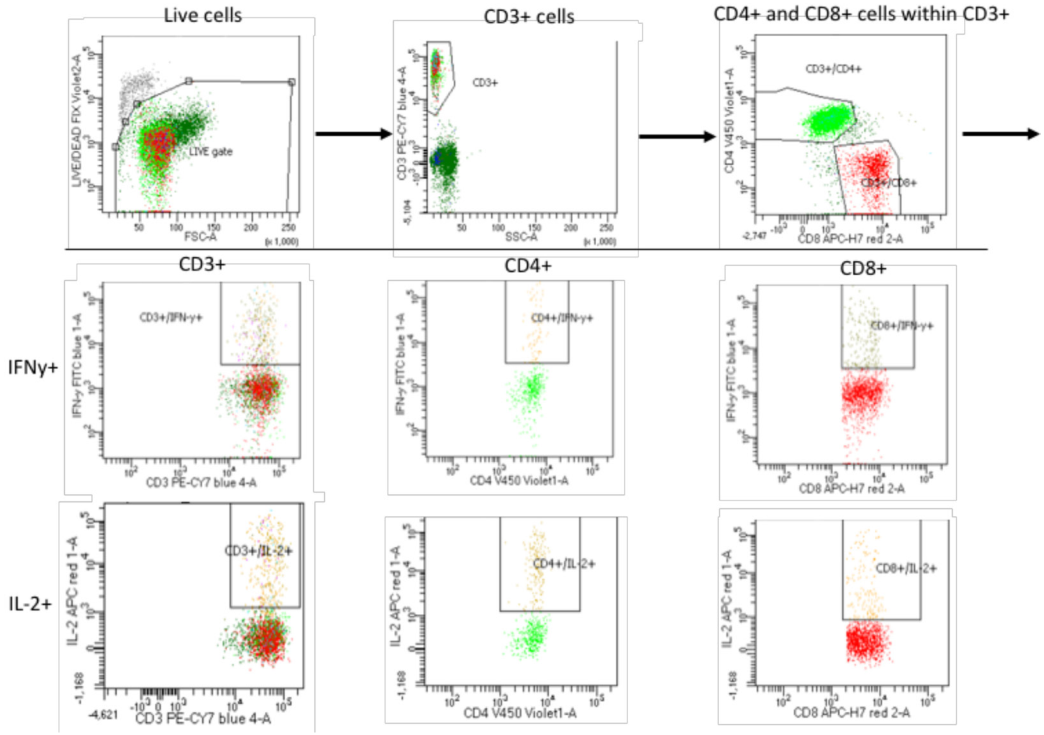

C Regulatory T-cells

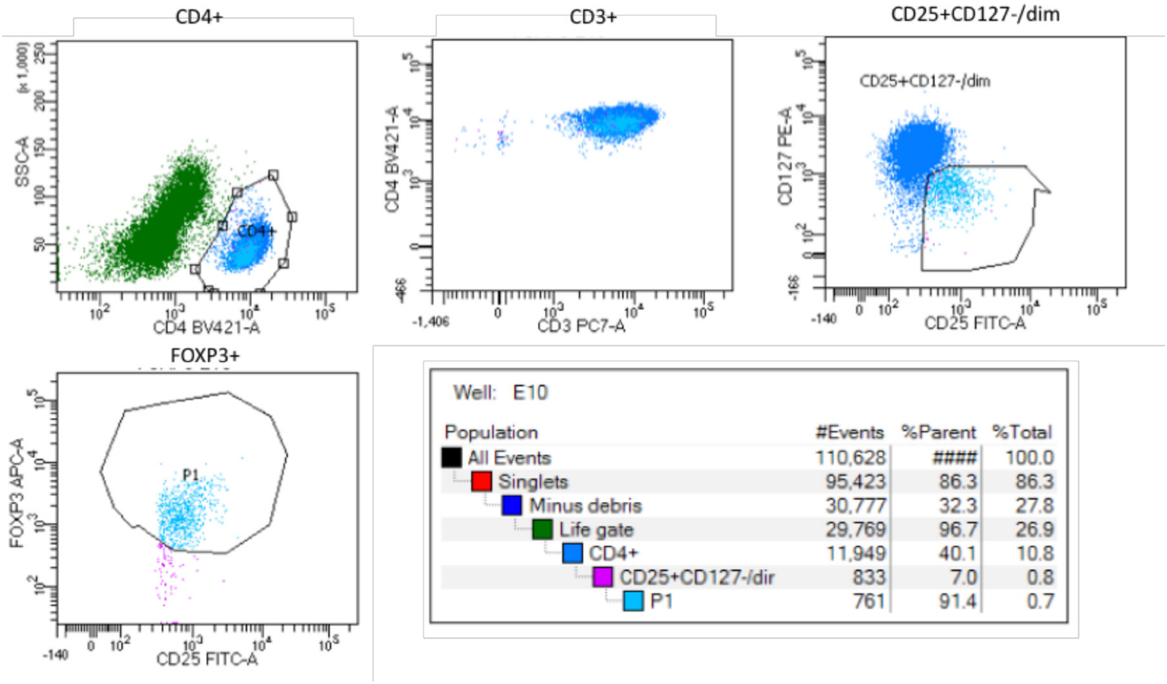

Population	#Events	%Parent	%Total
All Events	110,628	####	100.0
Singlets	95,423	86.3	86.3
Minus debris	30,777	32.3	27.8
Life gate	29,769	96.7	26.9
CD4+	11,949	40.1	10.8
CD25+CD127-/dir	833	7.0	0.8
P1	761	91.4	0.7

Supplementary Figure 1: Representative flow cytometry plots showing the gating strategy to detect different lymphocyte subsets. (**A**) Activated T-cells, defined as the percentage of T-cells expressing HLA-DR. CD3+/CD8- cells were considered CD4+ T-cells. (**B**) IFNγ and IL-2 positive T-cells. Cytokine production (IL-2 and IFNγ) of T-cells was measured after stimulating PBMCs with CD3/CD28 Human T-activator Beads (Dynabeads®) in a 1:1 ratio for 5 hours at 37° C in the presence of an inhibitor of intracellular protein transport (Brefeldin A, eBioscience). (**C**) Regulatory T-cells were defined as CD3+/CD4+/CD25+/CD127-/dim. FOXP3 intracellular staining was used to confirm the FOXP3 positivity of our defined regulatory T-cells. The FOXP3/Transcription Factor Staining Buffer Set (eBioscience) was used for the fixation and intracellular staining according to the manufacturer's protocol. The LIVE/DEAD® Fixable Dead Cell Staining kit (Thermofisher Scientific) was used to determine the viability of the cells prior to the fixation and permeabilization and subsequent intracellular staining.

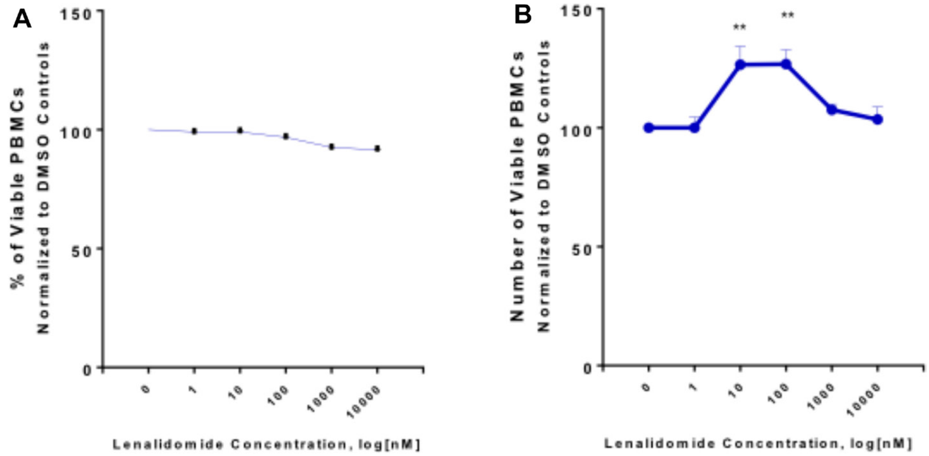

Supplementary Figure 2: Effect of lenalidomide on viability of PBMCs. Following DMSO or lenalidomide treatment and anti-CD3 stimulation for 72 hours, and prior to co-culture, viability of PBMCs was determined by flow cytometry. After 2 washes with PBS, these PBMCs were stained with Annexin-V (BD Biosciences) and To-Pro3 (Invitrogen) according to manufacturer's protocol, and CountBright™ Absolute Counting Beads (ThermoFisher) were added for absolute cell count. Live PBMCs were gated as Annexin-Vand Topro- population, and enumerated by the counting beads according to manufacturer's protocol. (**A**) Percent viable PBMCs of total PBMCs was plotted ($n = 4$). (**B**) Viable PBMCs were enumerated with Countbright™ absolute counting beads according to manufacturer's protocol ($n = 4$). *P*-values were calculated using one-way ANOVA, $^{**}P < 0.01$.
